# Madagascar's EPI vaccine programs: A systematic review uncovering the role of a child's sex and other barriers to vaccination

**DOI:** 10.3389/fpubh.2022.995788

**Published:** 2022-09-16

**Authors:** Emma Hahesy, Ligia Maria Cruz-Espinoza, Gabriel Nyirenda, Birkneh Tilahun Tadesse, Jerome H. Kim, Florian Marks, Raphael Rakotozandrindrainy, Wibke Wetzker, Andrea Haselbeck

**Affiliations:** ^1^Bowdoin College, Brunswick, ME, United States; ^2^International Vaccine Institute, Seoul, South Korea; ^3^University of Antananarivo, Antananarivo, Madagascar; ^4^Cambridge Institute of Therapeutic Immunology and Infectious Disease, University of Cambridge School of Clinical Medicine, Cambridge, United Kingdom; ^5^Jena University Hospital, Jena, Germany

**Keywords:** vaccines, EPI, Madagascar, sex differences, childhood immunization, vaccine uptake

## Abstract

**Background:**

Immunizations are one of the most effective tools a community can use to increase overall health and decrease the burden of vaccine-preventable diseases. Nevertheless, socioeconomic status, geographical location, education, and a child's sex have been identified as contributing to inequities in vaccine uptake in low- and middle-income countries (LMICs). Madagascar follows the World Health Organization's Extended Programme on Immunization (EPI) schedule, yet vaccine distribution remains highly inequitable throughout the country. This systematic review sought to understand the differences in EPI vaccine uptake between boys and girls in Madagascar.

**Methods:**

A systematic literature search was conducted in August 2021 through MEDLINE, the Cochrane Library, Global Index Medicus, and Google Scholar to identify articles reporting sex-disaggregated vaccination rates in Malagasy children. Gray literature was also searched for relevant data. All peer-reviewed articles reporting sex-disaggregated data on childhood immunizations in Madagascar were eligible for inclusion. Risk of bias was assessed using a tool designed for use in systematic reviews. Data extraction was conducted with a pre-defined data extraction tool. Sex-disaggregated data were synthesized to understand the impact of a child's sex on vaccination status.

**Findings:**

The systematic search identified 585 articles of which a total of three studies were included in the final data synthesis. One additional publication was included from the gray literature search. Data from included articles were heterogeneous and, overall, indicated similar vaccination rates in boys and girls. Three of the four articles reported slightly higher vaccination rates in girls than in boys. A meta-analysis was not conducted due to the heterogeneity of included data. Six additional barriers to immunization were identified: socioeconomic status, mother's education, geographic location, supply chain issues, father's education, number of children in the household, and media access.

**Interpretation:**

The systematic review revealed the scarcity of available sex-stratified immunization data for Malagasy children. The evidence available was limited and heterogeneous, preventing researchers from conclusively confirming or denying differences in vaccine uptake based on sex. The low vaccination rates and additional barriers identified here indicate a need for increased focus on addressing the specific obstacles to vaccination in Madagascar. A more comprehensive assessment of sex-disaggregated vaccination status of Malagasy children and its relationship with such additional obstacles is recommended. Further investigation of potential differences in vaccination status will allow for the effective implementation of strategies to expand vaccine coverage in Madagascar equitably.

**Funding and registration:**

AH, BT, FM, GN, and RR are supported by a grant from the Bill and Melinda Gates Foundation (grant number: OPP1205877). The review protocol is registered in the Prospective Register of Systematic Reviews (PROSPERO ID: CRD42021265000).

## Introduction

Vaccinations are a reliable and cost-effective way to decrease the burden of disease and improve the overall health of a community (1). Every year, immunizations save 2 to 3 million lives, and are one of the most effective ways to reduce global health inequities (2, 3). Despite global improvements in childhood immunization over the past decade, disparities in vaccination status persist within and across countries (4). Particularly in low- and middle-income countries (LMICs), differences in vaccine coverage exist based on one's socioeconomic status, geographical location, education level, and sex (5).

LMICs often exhibit low vaccine coverage rates relative to other countries (6). A majority of the global population of children with an incomplete vaccination series reside in LMICs (7). Several factors contribute to low rates of vaccine uptake in LMICs, including a lack of political support for vaccination campaigns, greater resource allocation to other health issues, and poor education and awareness about vaccines among healthcare workers and parents, specifically mothers (8–10). The lack of education about the benefits of immunization and risks of disease in LMICs further limits vaccine uptake, leading individuals to believe that immunization has a high cost-benefit ratio (8). Furthermore, insufficient human resources, lack of computerized vaccination registries and efficient communication systems with patients, and frequent issues with cold supply chains negatively impact vaccination rates in LMICs (9, 11).

Madagascar, an island country in Sub-Saharan Africa home to roughly 25 million people, has been identified as having one of the largest disparities in immunization rates in the world (5). Since 1976, Madagascar has followed the World Health Organization's (WHO) Expanded Programme on Immunization (EPI) to create a vaccine schedule (12). Recent evidence shows that immunization rates remain low, with WHO reporting MCV and DTP3 coverage at 69% and 79% in 2019 (13).

One 2010 WHO report identified a significant difference in childhood vaccination status based on sex in Madagascar, with girls more likely than boys to be fully vaccinated (14). Additionally, in Moramanga, Madagascar, there is a vast disparity in risk of death by age 15, at 58.4% for girls and 77.6% for boys, further indicating potential differential healthcare access between sexes in Madagascar (12). Sex differences in vaccination status have been identified in several other countries in Sub-Saharan Africa such as Malawi, Tanzania, and Namibia (14), demonstrating the existence of sex differences in immunization in other SSA countries.

Although imperative to understanding potential differences in vaccine uptake between boys and girls, research specifically identifying and quantifying the association between a child's sex and immunization uptake in Madagascar is limited (15). The purpose of this review was to assess the association between a child's sex and vaccination rates among children eligible for routine immunization programs, determine the availability of sex-disaggregated vaccination data, and identify other obstacles to vaccination in Madagascar.

## Methods

### Eligibility criteria and search strategy

All peer-reviewed, published articles including original research, editorials, short reports, clinical trials, and commentaries reporting sex-disaggregated data on childhood immunizations in Madagascar were eligible for inclusion. Pre-prints identified through the systematic search were not considered for inclusion. Articles were restricted to those in English and French, and no restrictions were put on the year of publication or study design.

This systematic review followed the PRISMA (Preferred Reporting Items for Systematic Review and Meta-Analyses) guidelines and the protocol has been published in the International Prospective Register of Systematic Reviews (PROSPERO, ID: CRD42021265000) and can be accessed through the following link: https://www.crd.york.ac.uk/prospero/display_record.php?RecordID=265000 (16). The protocol was amended after submission to include additional obstacles to immunization as a secondary outcome of interest, and such changes are reflected in the published protocol.

The population, intervention, comparison, and outcomes (PICO) framework was used to determine article eligibility. Under this framework, the population was defined as children eligible for vaccination through routine immunization programs in Madagascar, intervention as routine immunization programs in Madagascar, comparison as comparing vaccine coverage between sexes, and outcome as sex-disaggregated vaccination data (including rates, proportions, and other measurements) in children eligible for immunization in Madagascar.

MEDLINE (*via* PubMed), Cochrane Library, Global Index Medicus (GIM), and Google Scholar were searched in the 1st week of June 2021 to identify relevant articles. All search results were restricted to those in English and French to ensure that the results fit the eligibility criteria. MeSH terms were used in MEDLINE and the same keywords were used for the search in the other three libraries (Supplementary Table 1).

**Figure 1:**
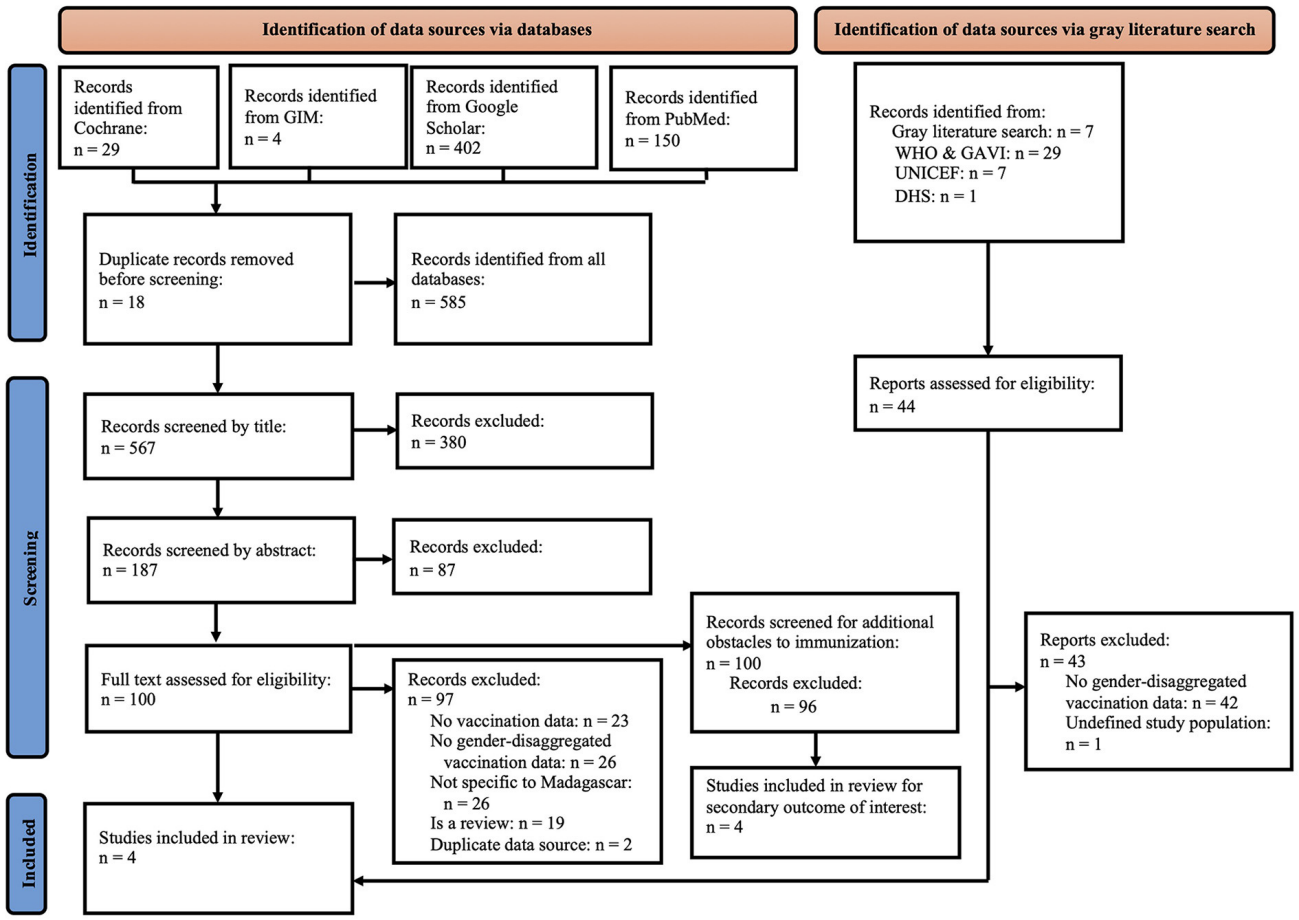
PRISMA flowchart showing the number of articles screened by title, abstract, and full-text for the systematic and gray literature search and illustrating the inclusion and exclusion of articles as well as the reasons for exclusion. WHO and GAVI are listed together under gray literature search as WHO presented data from GAVI. DHS, Demographic Health Survey; GAVI, Gavi the Vaccine Alliance; GIM, Global Index Medicus; WHO, World Health Organization.

Reviewers EH and AH independently screened all articles identified from MEDLINE, Cochrane Library, GIM, and Google Scholar in a three-step process wherein the title, abstract, and full-text were reviewed to determine eligibility for data extraction. EH, AH, and LC worked together to determine which articles would be included for data extraction and resolve any disagreements. A PRISMA flowchart detailing this process is presented in Figure 1. Data extraction was conducted by EH using a pre-defined data extraction tool on Microsoft Excel (Supplementary Table 2). The reference lists of identified articles included for full-text screening were hand-searched by EH to identify additional relevant articles.

The WHO, Gavi the Vaccine Alliance (GAVI) and Malagasy Ministry of Health websites were hand-searched for relevant information. Documents related to health and vaccination in Madagascar or containing information from demographic surveys were screened. A supplementary online search using the Google general search engine was conducted to identify additional data sources not previously identified (keywords: gender + disaggregated + vaccination + data + Madagascar). These searches were conducted in the 1st week of August 2021.

The authors contacted the Ministry of Health in Madagascar to access governmental immunization statistics relevant to this review. No documents had been shared at the time of this publication.

### Risk of bias assessment

The Appraisal tool for Cross-Sectional Studies (AXIS tool) was used to assess the risk of bias in included articles. This tool assesses the clarity of reporting, study design, data collection and sample selection, consistency of results, recognition of limitations, and influence of conflicts of interest. Developed in 2016, this tool is recommended for cross-sectional studies and systematic reviews and was determined to be most relevant to this research (17, 18).

The AXIS tool consists of 20 questions that researchers divided into the six categories listed above for simplicity (17). A grade of low, moderate, or high was given to each category in an article. A low grade indicates that risk of bias was found in a majority of the questions within the category. A moderate grade means that some, but not a majority, of the questions within the category indicated a risk of bias. A high grade means that there was no indication of a risk of bias. Supplementary Table 3 provides information about the risk of bias categories created and specific quality measures. To determine the overall risk of bias judgement for an article, the categories were assessed together, and a judgement was made based on the same metric of low, moderate, and high risk of bias. Any studies determined to be of low overall quality were to be excluded from this review (Table 1).

**Table 1:** Risk of bias assessment results after using the AXIS tool.

**First author, year of publication**	**Clarity of reporting**	**Study design**	**Data collection and sample selection**	**Internal consistency of results**	**Recognition of limitations**	**No conflicts of interest**	**Overall quality**
Bosch-Capblanch, 2012 (17)	Moderate	High	High	High	High	High	High
Hill, 1995 (18)	High	High	Moderate	High	High	Low	Moderate
GAVI, 2019 (13)	High	High	High	High	N/A	High	High
Restrepo-Méndez, 2016 (16)	High	High	High	High	High	High	High

A high grade indicates no risk of bias in any question within the category. A moderate grade indicates that risk of bias was found in some, but not a majority of the questions within the category. A low grade indicates that risk of bias was found in a majority of the questions within a category. Three included studies demonstrate a high overall quality while one demonstrates a moderate overall quality. No studies had to be excluded for low quality.

### Data synthesis and presentation

**Figure 2:**
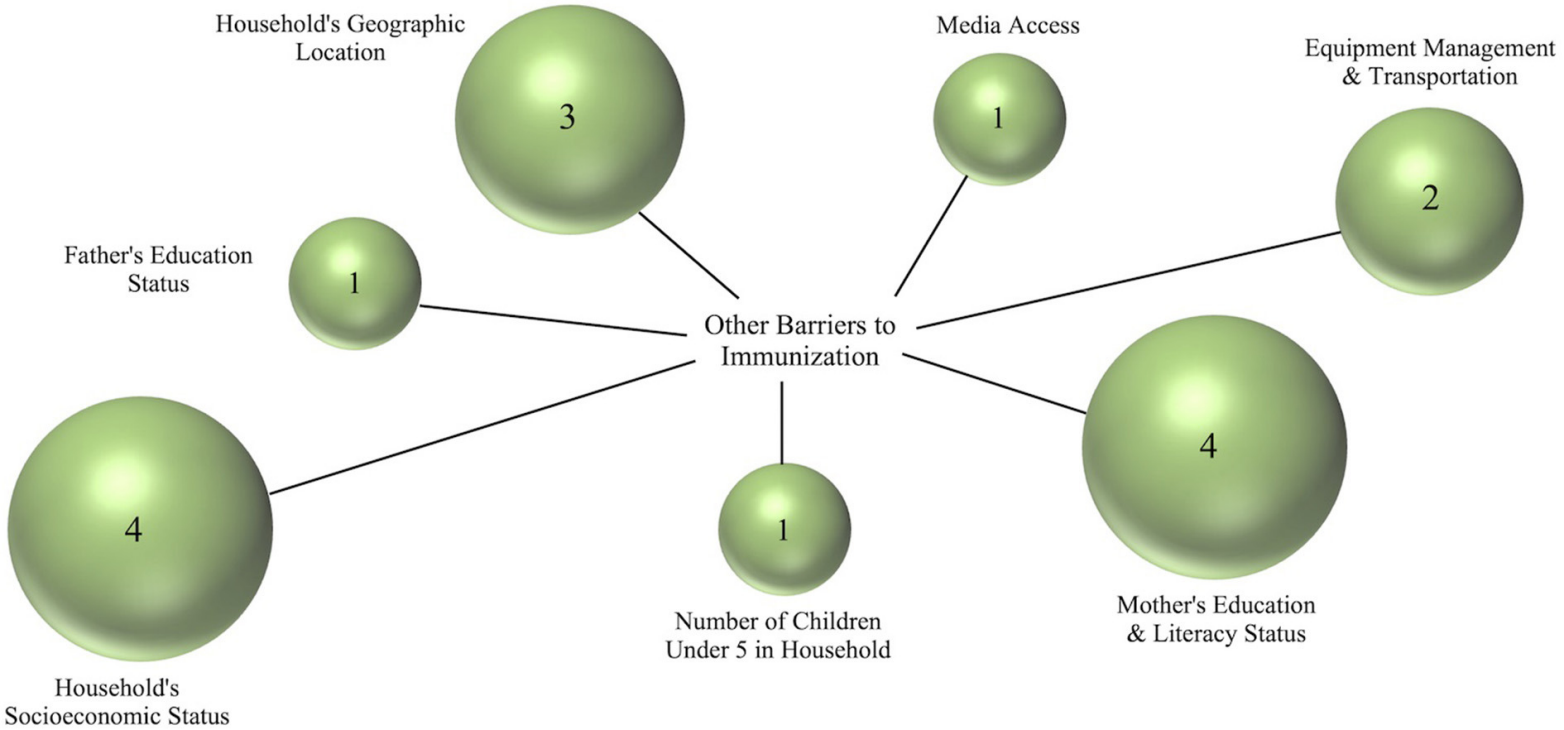
Frequency of reported barriers to immunization other than sex. Such factors were qualitatively assessed and presented based on how many articles included in the full-text screening mentioned each factor. Bubble size corresponds to the number of articles that mentioned each factor, as does the number inside each bubble. Household's socioeconomic status and female education/literacy status were the two factors listed most frequently in such articles. Media access refers to an individual's access to information (including information about vaccinations) via the internet; Equipment management and transportation refers to managing refrigeration, gasoline, and the transportation of materials used for immunizations to health centers.

Immunization data were considered eligible for synthesis if it was sex-disaggregated and specific to children in Madagascar, as the primary outcome of interest was sex-disaggregated vaccination data in Malagasy children. Included effect measures were odds ratios and percentages of girls and boys vaccinated. All articles identified during the search were screened and timeframe restrictions were not used. To better understand vaccine distribution in Madagascar, researchers also noted additional barriers to immunization in articles already presenting sex-disaggregated vaccination data. Such data were reported qualitatively as a secondary outcome of interest in Figure 2.

A meta-analysis could not be performed on the type of data collected in this review given the low number of articles included and the different measures used by the authors to present sex-disaggregated data. Data about the primary outcome of interest, sex-disaggregated vaccination data, from all sources are presented in Table 2. Articles presenting vaccination data in the form of a percentage are listed first as they allow for a clear comparison between sexes. The age of children studied, vaccine type, data source, and year of data collection were also included in Table 2.

**Table 2:** Sex-disaggregated vaccination data reported in included articles.

**First author/Publishing organization**	**Year of publication**	**Data source**	**Year of data collection**	**Age of study population (months)**	**Vaccine type**	**Sex-disaggregated vaccination data**		**Sex with higher vaccination rate**
						**Boys**	**Girls**	
Restrepo-Méndez (16)	2016	DHS	2008	12–23	BCG, DPT3, polio3, measles	61.4%^*^	61.9%^*^	Girls
GAVI (13)	2019	MICS	2018	12–23	BCG, DTP3, polio3, measles	42%^**^	40%^**^	Boys
Hill (18)	1995	DHS	1992	12–23	BCG, DPT3, polio3, measles	Difference between percentage of girls and boys vaccinated: 2.0**		Girls
Bosch-Capblanch (17)	2012	DHS	2004	12–59	BCG, DTP3, polio3, measles	Odds ratio of being unvaccinated: 0.77^***^		Girls

Data is presented in the form of percentage immunized, differences in percentage immunized by sex, and the odds ratio of being unvaccinated. BCG, Bacillus Calmette-Guérin Vaccine; DHS, Demographic Health Survey; DPT3, Diphtheria, Pertussis, and Tetanus Three-Dose Vaccine; GAVI, Global Alliance for Vaccines and Immunizations; GIM, Global Index Medicus.

* Standard error of 2.0.

** No statistical significance reported.

*** Confidence interval of 0.60 to 1.00.

## Results

The systematic search of four libraries retrieved 585 results, 29 (49.6%) from the Cochrane library, four (6.8%) from GIM, 402 (68.7%) from Google Scholar, and 150 (25.6%) from PubMed. Following rounds of title and abstract screening, full-text screening was conducted for 100 remaining records, of which 96 were excluded. Of these 96 records, 23 (24.0%) were excluded for not reporting any vaccination data, 26 (27.0%) were excluded for not including data specific to Madagascar, 19 (19.8%) were reviews, two (3.4%) contained duplicated data sources, and one (1.0%) did not report data specific to children.

A total of 44 records were identified through the gray literature search. Of these, 42 (95.4%) were excluded because they did not contain sex-disaggregated vaccination data and one (2.3%) was excluded because it had an undefined study population. The remaining publication by GAVI (19) had an identical data source to an article previously identified and included by the systematic search by Mutua et al. (20). The GAVI source reported vaccination data in terms of percentages of children vaccinated as opposed to the slope index of inequality used in Mutua et al.'s study, so the GAVI source was included instead (Supplementary Table 4). In total, three articles from the systematic search and one database from GAVI were finally included for data extraction (Figure 1). All four included articles presented secondary data originally collected through DHS and MICS and all extracted data are publicly available.

Risk of bias assessments were conducted on the four included publications using the AXIS tool (21). Three studies had no risk of bias and were considered of high quality (19, 22, 23) and one study had a moderate risk of bias (24) (Table 1). The cross-sectional study conducted by Restrepo-Méndez et al. and the 2019 GAVI report did not demonstrate any risk of bias and thus are of high quality (19, 22). Bosch-Capblanch et al. did not include a consolidated methods section but was otherwise of high quality (23). Hill et al. had a small sample size which, although recognized and acknowledged by the researchers, detracts from the quality of the study. Additionally, Hill et al. did not explicitly declare a conflicts of interest statement. Although this could be attributed to a change in the reporting of conflicts of interest over time, as this is the oldest study included in the review, this publication received a moderate quality grade (24) (Table 1).

All publications reported coverage of the basic vaccines recommended through the EPI program: BCG, DTP3, Polio3, and Measles. Three of the publications reported vaccination rates for children 12–23 months old (19, 22, 24) and one for children 12–59 months old (23). Two publications measure the outcome of interest in the form of percentages of boys and girls vaccinated (19, 22); one publication as the difference between percentage of girls and boys vaccinated (24), and another publication used an odds ratio to illustrate the sex difference (23). Data sources for the publications included were the Demographic and Health Surveys (DHS) in 1992 (24), 2004 (23), and 2008 (22); and a Multiple Indicator Cluster Survey (MICS) in 2018 (19).

Three included studies reported higher vaccination in girls (22–24) and one reported higher vaccination in boys (19) (Table 2).

Among the 100 full-text articles assessed for reported additional obstacles to immunization, four publications mentioned obstacles other than sex (4, 5, 25, 26). Socioeconomic status, mother's literacy and education, geographic location, supply chain issues, father's education, number of children in the household, and media access were all found to impact immunization status in Madagascar. Three publications mentioned socioeconomic status (4, 5, 25), three mother's literacy and education (4, 5, 26), two geographic location (4, 5), two equipment management and transportation (4, 5), one father's education (4), one number of children in household (26), and one media access (26).

## Discussion

The evidence collected in this systematic review shows a minimal difference in reported vaccination rates between boys and girls (22–24). However, the scarcity of evidence and heterogeneous nature of the data limit our ability to conclusively confirm or deny the existence of sex-based disparities in Madagascar's vaccination program. Of the four included reports, one reports a statistically significant sex difference in vaccine uptake (23), one reports no difference (22), and two report a two percent difference in vaccine uptake between boys and girls but no measure of statistical significance (19, 24). These conflicting reports and the lack of overall data mean that additional sex-disaggregated data about the EPI vaccine uptake of children are required to confirm or deny sex-based disparities in vaccination status in Madagascar.

Sex differences in vaccine uptake have been previously identified in other African countries including Mozambique and the Democratic Republic of the Congo (20). Conversely, sex has also been found to have no impact on vaccine uptake in Nigeria, Ethiopia, and Kenya (27–29). Interestingly, one report found a statistically significant sex imbalance in vaccination in East Africa (20) despite additional reports claiming that sex did not influence vaccination status in East Africa (30) or Sub-Saharan African as a whole (31). Such inconsistencies may reflect the fact that local or regional disparities can be masked by regional or national averages (4) and the existence of countries with and without sex-based differences in vaccine uptake across Africa. Analyses of immunization trends in Africa thus indicate that the existence or lack of sex-based disparities in EPI vaccine uptake in Madagascar are both plausible, again highlighting a need for greater reporting of sex-disaggregated vaccination data.

Further demonstrating the importance of collecting and analyzing vaccination data based on individual demographic factors, we identified several other factors causing differential vaccine uptake in Madagascar including a household's socioeconomic status (4, 5, 25), geographical location (4, 5), mother's education and literacy status (4, 5, 26), and the management and transportation of immunization resources (4, 5). Such barriers in Madagascar reflect reports citing socioeconomic status (26, 30, 32) community wealth (30), parent's education (30, 32), and media access (30) as causes of differential vaccination uptake in other African countries. It is important to note that the articles screened for additional obstacles to vaccination were identified *via* the original search strategy focusing on a child's sex and vaccination, likely narrowing our results. Reports of additional barriers are intended to indicate the presence of other factors associated with vaccination status to provide ideas for future direction and focus rather than providing a comprehensive report.

The influence of maternal education level on vaccination status is of note and was found to strongly impact a child's vaccination status in Madagascar. A 2019 GAVI report found that 57% of children whose mothers had at least a secondary level of education were vaccinated compared to only 24% of children with uneducated mothers (19). Another study reported that children with educated mothers were 1.7 times more likely to be vaccinated than children with uneducated mothers (33). The increases in household wealth and decreases in fertility associated with educated mothers positively influence childhood survival rates in Madagascar, and we posit that such effects may account for the influence of mother's education over vaccination status as well (34). Further research to understand why female education status impacts a child's vaccination status in Madagascar is needed to confirm or deny these hypotheses.

Differences in vaccination rates between wealthy and poor or urban and rural households have also been identified (4, 5, 19, 25). Children in the affluent Itasy region of Madagascar are 3.4 times more likely to be vaccinated than those in the poorer and more remote Menabe region, revealing Madagascar's large regional and socioeconomic inequalities in vaccine uptake (5). Such socioeconomic and geographic differences in vaccination status reflect the obstacles that poorer, rural-dwelling Malagasy people face in accessing healthcare facilities. Unreliable transportation methods, poor roads, and rainy seasons that flood travel routes and render villages unreachable present considerable challenges to health care and vaccination access for rural residents (19, 35), negatively impacting vaccination status (4, 5). The management of resources needed for immunization such as refrigeration, gasoline, and the transportation of cold chain supplies is challenging in rural areas, making it hard to provide adequate resources for vaccinations (4). Furthermore, Madagascar only has 0.14 physicians for every 1,000 patients compared to a global average of 1.4 physicians, creating an additional barrier to healthcare access (36). We recommend taking an intersectional approach to assess whether such barriers identified here differentially affect boys and girls in Madagascar.

In addition to the notable scarcity in sex-disaggregated data and the lack of clear difference in vaccination rates by sex, this review also revealed a recent decrease in immunization rates in Madagascar. The latest comprehensive assessment published in 2019 by GAVI highlights a decrease in immunization in girls from 61.9% (22) to 40% (19) and in boys from 61.4% (22) to 42% (19) between 2008 and 2019. Effective vaccine coverage for the measles, a disease against which EPI vaccines provide protection, is estimated at 95% by WHO, indicating that Madagascar's vaccine coverage is at an alarming low level (37).

Notably, researchers identified stark differences in the reporting of vaccination coverage across sources, potentially explaining the seemingly low immunization rates reported in the 2019 GAVI assessment (19). One WHO publication reported rates of EPI vaccination ranging from 62% coverage for MCV1 to 81% coverage for DTP1 in 2018. This report notes the difference of up to 20% between coverage estimates provided by WHO-UNICEF, the Malagasy government, and administrative reports (38). Another WHO publication reported 2018 coverage for MCV1 at 85% and DTP1 at 97%, again demonstrating a vast discrepancy in immunization data collection and reporting (13). The inconsistencies in the reporting of immunization coverage are troubling and should be of concern to data collection organizations and the Malagasy government.

Regardless of these discrepancies in estimated vaccine coverage levels, vaccination coverage in Madagascar appears to be far below the levels needed to confer herd immunity and prevent outbreaks (37). In 2018, a measles outbreak struck Antananarivo, Madagascar's capital, and extended rapidly to all 22 regions in Madagascar due to low vaccination coverage estimated at 60% by WHO-UNICEF (39). Low immunization rates remain a critical threat to the health of citizens and the decrease in vaccination rates noted in this review is a cause for concern. The COVID-19 pandemic has led to further decreases in vaccination rates (40). As of October 2021, no global datasets had published sex-disaggregated data on COVID-19 vaccine uptake despite existing reports indicating that women in LMICs are less likely to receive COVID-19 vaccines than men (41). The impact of the COVID-19 pandemic on EPI vaccination programs must continue to be monitored closely, and the role of a child's sex must not be forgotten.

One limitation and simultaneously a finding of this research is the lack of sex-disaggregated vaccination data collected in demographic surveys. Many reports contain information about vaccination rates in Madagascar, but few are sex-disaggregated, limiting our ability to assess sex differences in vaccine uptake. Another limitation is the heterogeneous nature of the data collected, with many sources reporting vaccination data differently and preventing researchers from conducting a meta-analysis. A recent scoping review has highlighted the importance of high-quality vaccination data and the current paucity of such in LMICs (42). Our review supports this finding and underlines the urge for consistent methodology in data collection and methods of analyses to allow comparison on relevant levels and inform future adaptation of vaccine distribution strategies.

The lack of sex-disaggregated data identified in this review reveals a gap in current understandings of how a child's sex affects health status. The scarcity of available data not only precludes researchers from determining whether sex influences vaccine uptake in Madagascar but indicates an insufficient understanding of how sex may interact with the additional barriers identified here. These gaps in current understandings of vaccine uptake are concerning as they may allow inequities to remain unaddressed. A more comprehensive assessment of the vaccination status of Malagasy children based on defined variables including sex and household information is desirable to confirm potential demographic factors contributing to inequity in vaccine uptake. A thorough and consistent collection of sex-disaggregated vaccination data in Madagascar would allow for a monitoring of the impact of sex on vaccination status over time and intersectional analyses to understand the relationship between sex and other barriers to vaccination (43). Further, in the event of large-scale disease outbreaks such as the 2018 measles outbreak and the ongoing COVID-19 pandemic, any potential sex imbalances in vaccination could be readily identified and corrected.

## Conclusion and recommendations

This is the first systematic review to look at sex differences in EPI vaccination in Madagascar to our knowledge. The lack of sex-disaggregated vaccination data highlights an unmet need and gap in current understandings of vaccine uptake. Interestingly, socioeconomic status, geographic location, mother's education status, and immunization equipment management were all identified as causes of vaccine inequity in Madagascar, directing attention away from sex and toward overcoming other barriers. We recommend future analyses to weigh the relative impact of such factors and inform interventions designed to improve vaccine coverage.

Of note is our finding that vaccination rates in Madagascar have drastically decreased over the past 15 years, with an additional decrease due to the COVID-19 pandemic. Future monitoring of sex-disaggregated vaccination data would reveal whether similar drastic changes in vaccination rates differentially affect boys and girls. We suggest that increasing the vaccination rate in Madagascar is of the utmost importance. During this process, specific attention should be paid to closing the gaps that are identified between rural and urban, rich and poor, and educated and non-educated populations while maintaining an awareness of the potential for sex inequity in vaccine uptake in the future *via* the collection of sex-disaggregated vaccination data during routine EPI vaccinations.

Implementing interventions to expand vaccination coverage across LMICs is recommended. Suggested interventions include outreach campaigns (going to houses, schools, markets, and other places where people gather), healthcare education (particularly community-based), and home visits. Such interventions are intended to decrease the reliance on self-presentation by individuals (mothers, in most cases) to the healthcare facility in order to complete EPI in children. Similar strategies have previously been used to improve vaccination rates in LMICs (7). In particular, we recommend the continuation and expansion of National Immunization Days (NIDs), Subnational Immunization Days (SNIDs), Child Health Days, and Vaccination Weeks (VWs) in Madagascar and similar settings. These programs involve establishing additional outreach locations for vaccinations, knocking on doors to vaccinate people, hosting “mother and child health weeks” at primary care facilities, and catching children who missed or are late on a vaccine dose, and have previously helped improve vaccination rates in LMICs (44–47).

We additionally recommend a focus on implementing and expanding media coverage, mass informational campaigns, and direct physician to patient communication about vaccination and the dangers of vaccine-preventable diseases in LMICs. Increased public education and awareness of the benefits of immunization have been identified as useful interventions to increase political support, reduce sociocultural barriers, and improve community attitudes toward immunization (8, 9). Health and government organizations designing informational campaigns must take into account the literacy and education level of the target audience (8) as well as the specific barriers to immunization in a community to ensure the campaign is effective and well-received. We also urge the importance of increasing the quality of data collection and ensuring external consistency of immunization data to allow for accurate future analysis. To do so, potential weaknesses in the surveillance of vaccine uptake data should be assessed and strengthened accordingly.

Directing attention toward new vaccine delivery technologies such as vaccine-containing microarray patches (VMAPs), could have the potential to reduce barriers to immunization in LMICs. With an intradermal delivery method, higher thermo stability, and fewer training requirements for healthcare workers than ordinary vaccines, VMAPs are favorable for delivery to rural areas and regions in conflict (48, 49) Research and development of VMAPs for diseases including measles, rubella, HPV, and influenza are currently underway (49, 50) and should be monitored to determine whether they are suitable for implementation in Madagascar and other LMICs.

The recommendations outlined in this review and our suggestion to increase the collection of sex-disaggregated vaccination data have the potential to improve vaccination rates, attitudes about vaccines, and understandings of how demographic factors impact vaccination status in many LMICs. Greater understandings of the barriers to vaccination and existing inequities will allow for the implementation of specific interventions to target such obstacles. Doing so will ensure that LMICs increase their vaccination rates in a manner that is equitable across demographic groups.

## Data availability statement

The original contributions presented in the study are included in the article/Supplementary files, further inquiries can be directed to the corresponding author/s.

## Author contributions

EH, AH, and LC-E contributed to the study design, methodology, article screening, and data interpretation. EH conducted the literature search and collected data. AH and LC-E provided supervision. GN and RR attempted to access immunization data from the Malagasy government. All authors contributed to manuscript revision, read, and approved the submitted version.

## Funding

AH, BT, FM, GN, and RR are supported by a grant from the Bill and Melinda Gates Foundation (Grant Number: OPP1205877).

## Conflict of interest

The authors declare that the research was conducted in the absence of any commercial or financial relationships that could be construed as a potential conflict of interest.

## Publisher's note

All claims expressed in this article are solely those of the authors and do not necessarily represent those of their affiliated organizations, or those of the publisher, the editors and the reviewers. Any product that may be evaluated in this article, or claim that may be made by its manufacturer, is not guaranteed or endorsed by the publisher.
