# Supplementary material for: Madagascar's EPI vaccine programs: A systematic review uncovering the role of a child's sex and other barriers to vaccination
Source: Front Public Health. 2022 Sep 16;10:995788. doi: 10.3389/fpubh.2022.995788 (PMC9523513; doi:10.3389/fpubh.2022.995788)
Supplement: Supplementary file 1 [file Table_1.DOCX]

**Supplementary Table 1.** Detailed search terms used in literature search of MEDLINE, the Cochrane Library, Google Scholar, and Global Index Medicus.

|  | **1** | **BO** | **2** | **BO** | **3** | **BO** | **4** |
| --- | --- | --- | --- | --- | --- | --- | --- |
| **Concepts** | Childhood Immunization |  | Access |  | Sex Differences |  | Location |
| **Keywords** | *Vaccine/ Immunization/ Health/ Vaccination Coverage*/ Childhood Immunization* | **AND** | *Healthcare Disparities*/*  *Rate/*  *Access/ Distribution/ Intervention/ Program* | **AND** | *Gender Equity*/*  *Gender/*  *Male/*  *Female* | **AND** | *Madagascar** |

**Included as MESH term in MEDLINE search*

***BO: Boolean Operator*
