# Supplementary material for: Madagascar's EPI vaccine programs: A systematic review uncovering the role of a child's sex and other barriers to vaccination
Source: Front Public Health. 2022 Sep 16;10:995788. doi: 10.3389/fpubh.2022.995788 (PMC9523513; doi:10.3389/fpubh.2022.995788)
Supplement: Supplementary file 2 [file Table_2.DOCX]

**Supplementary Table 2.**Data extraction tool developed by authors and used to extract data from identified articles.

| ***Category*** | **Information Extracted** |
| --- | --- |
| *General* | Reference No. |
|  | First Author |
|  | Year of Publication |
|  | Title |
|  | Type of Publication |
| *Methods* | Objective of Study/Research Question |
|  | Study Design |
|  | Recruitment Procedure/Sampling Process |
|  | Study/Project Time Period |
|  | Source of Vaccination Data |
| *Population* | Study Population Including Age Group |
|  | Place/Country |
|  | Setting (rural/urban) |
| *Vaccine Details* | Vaccine Type |
|  | Vaccine Doses |
| *Results* | Outcome: Overall Vaccination Data |
|  | Vaccination Data Stratified by Sex |
|  | Analysis and Commentary on Vaccines/Sex |
|  | Reported Obstacles to Immunization |
|  | Commentary (including reported limitations) |
|  | Recommendations for Future |
|  | Study Conclusions |
| *Other* | Limitations Identified by Screening Person |
|  | Assessment (grade given by risk of bias assessment) |
|  | Additional Comments |
