# Supplementary material for: Madagascar's EPI vaccine programs: A systematic review uncovering the role of a child's sex and other barriers to vaccination
Source: Front Public Health. 2022 Sep 16;10:995788. doi: 10.3389/fpubh.2022.995788 (PMC9523513; doi:10.3389/fpubh.2022.995788)
Supplement: Supplementary file 3 [file Table_3.DOCX]

**Supplementary Table 3.**Categories used to assess risk of bias using the AXIS tool. Questions were summarized in different categories to aid in the assessment of risk of bias.

| *Risk of Bias Category* | **Question** |
| --- | --- |
| *Clarity of Reporting* | Were the aims/objectives of the study clear? |
|  | Was the target/reference population clearly defined? |
|  | Is it clear what was used to determine statistical significance and/or precision estimates? |
|  | Were the methods sufficiently described to enable them to be repeated? |
|  | Were the basic data adequately described? |
|  | Were the results presented for all the analyses described in the methods? |
|  | Were the authors’ discussions and conclusions justified by the results? |
| *Study Design* | Was the study design appropriate for the stated aims? |
|  | Was the sample size justified? |
|  | Were the risk factor and outcome variables measured appropriate to the aims of the study? |
|  | Were the risk factor and outcome variables measured correctly using instruments/measurements that had been trialed, piloted, or published recently? |
| *Data Collection & Sample Selection* | Was the sample frame taken from an appropriate population base so that it closely represented the target/reference population under investigation? |
|  | Was the selection process likely to select subject/participants that were representative of the target/reference population under investigation? |
|  | Was ethical approval or consent of participants attained? |
| *Consistency of Results* | Were the results internally consistent? |
| *Recognition of Limitations* | Were the limitations of the study discussed? |
| *Conflicts of Interest* | Were there any funding sources or conflicts of interest that may affect the authors’ interpretation of the results? |
