# Supplementary material for: Madagascar's EPI vaccine programs: A systematic review uncovering the role of a child's sex and other barriers to vaccination
Source: Front Public Health. 2022 Sep 16;10:995788. doi: 10.3389/fpubh.2022.995788 (PMC9523513; doi:10.3389/fpubh.2022.995788)
Supplement: Supplementary file 4 [file Table_4.DOCX]

**Supplementary Table 4.** Articles excluded by full text and reason for exclusion.

| **ID** | **Title** | **Reason for Exclusion** |
| --- | --- | --- |
| 1 | Rural-urban disparities in missed opportunities for vaccination in sub-Saharan Africa: a multi-country decomposition analyses | No vaccination data specific to Madagascar |
| 2 | The path to longer and healthier lives for all Africans by 2030: the Lancet Commission on the future of health in sub-Saharan Africa | No sex-disaggregated vaccination data specific to Madagascar |
| 3 | Evaluation of invalid vaccine doses in 31 countries of the WHO African Region | Is a review |
| 4 | Mass vaccination campaigns to eradicate poliomyelitis in Madagascar: oral poliovirus vaccine increased immunity of children who missed routine programme | No sex-disaggregated vaccination data |
| 5 | Wild poliovirus circulation among healthy children immunized with oral polio vaccine in Antananarivo, Madagascar | No sex-disaggregated vaccination data |
| 6 | Monitoring equity in vaccination coverage: a systematic analysis of demographic and health surveys from 45 Gavi-supported countries | Is a review |
| 7 | Country-level predictors of vaccination coverage and inequalities in Gavi-supported countries | Is a review |
| 8 | Madagascar's vulnerable children | No sex-disaggregated vaccination data |
| 9 | Barriers to childhood immunization in sub-Saharan Africa: a systematic review | Is a review |
| 10 | Madagascar's health challenges | No sex-disaggregated vaccination data |
| 11 | Equity in maternal, newborn, and child health interventions in Countdown to 2015: a retrospective review of survey data from 54 countries | Is a review |
| 12 | Comparative performance of private and public healthcare systems in low-and middle-income countries: a systematic review | Only included in full text because could not identify abstract |
| 13 | Socio-economic inequalities in child stunting reduction in sub-Saharan Africa | Only included in full text because could not identify abstract |
| 14 | Countdown to 2015 decade report (2000–10): taking stock of maternal, newborn, and child survival | No sex-disaggregated vaccination data specific to Madagascar |
| 15 | Letter from Madagascar | No vaccination data |
| 16 | [Tuberculosis in children in Madagascar. 122 cases observed at the Soavinandriana-Antananarivo Hospital Center] | Could not access full text |
| 17 | Hepatitis B virus infection in general population in Madagascar: evidence for different epidemiological patterns in urban and in rural areas | No sex-disaggregated vaccination data |
| 18 | Human papilloma virus vaccination: impact and recommendations across the world | Is a review |
| 19 | Unvaccinated children in years of increasing coverage: how many and who are they? Evidence from 96 low‐and middle‐income countries | Is a review |
| 20 | Child survival in East Africa: The impact of preventive health care | Is a review |
| 21 | Female Education in Sub-Saharan Africa: The Key to Development? | Is a review |
| 22 | Measuring coverage in MNCH: new findings, new strategies, and recommendations for action | No vaccination data specific to Madagascar |
| 23 | Countdown to 2015: tracking intervention coverage for child survival | No sex-disaggregated vaccination data specific to Madagascar |
| 24 | A qualitative evaluation of health care in the Maroantsetra region of Madagascar | No sex-disaggregated vaccination data specific to Madagascar |
| 25 | Human papillomavirus prevalence and type-specific distribution of high- and low-risk genotypes among Malagasy women living in urban and rural areas | No sex-disaggregated vaccination data specific to Madagascar |
| 26 | Immunization, urbanization and slums–a systematic review of factors and interventions | Is a review |
| 27 | A prediction model of childhood immunization rates | Only included in full text because could not identify abstract |
| 28 | Pro-equity immunization and health systems strengthening strategies in select Gavi-supported countries | No sex-disaggregated vaccination data |
| 29 | Forecasted trends in vaccination coverage and correlations with socioeconomic factors: a global time-series analysis over 30 years | No sex-disaggregated vaccination data specific to Madagascar |
| 30 | Acceptability of coupling intermittent preventive treatment in infants with the expanded programme on immunization in three francophone countries in Africa | No sex-disaggregated vaccination data specific to Madagascar |
| 31 | Resource allocation for health equity: issues and methods | No sex-disaggregated vaccination data specific to Madagascar |
| 32 | Introducing auto-disable syringes to the national immunization programme in Madagascar | No vaccination data specific to children |
| 33 | Girl child marriage as a risk factor for early childhood development and stunting | No vaccination data |
| 34 | Assessing trends in the content of maternal and child care following a health system strengthening initiative in rural Madagascar: A longitudinal cohort study | No sex-disaggregated vaccination data |
| 35 | Impact of Childhood Vaccinations Suspended Due to COVID-19 | Is an infographic |
| 36 | Sex and gender differences in the outcomes of vaccination over the life course | No sex-disaggregated vaccination data specific to Madagascar |
| 37 | Gender imbalance in infant mortality: a cross-national study of social structure and female infanticide | No sex-disaggregated vaccination data specific to Madagascar |
| 38 | Poverty reduction and Millennium Development Goals: recognizing population, health, and environment linkages in rural Madagascar | No vaccination data |
| 39 | Charting the evolution of approaches employed by the Global Alliance for Vaccines and Immunizations (GAVI) to address inequities in access to immunization: a systematic qualitative review of GAVI policies, strategies and resource allocation mechanisms through an equity lens (1999–2014) | Is a review |
| 40 | In Madagascar, Use Of Health Care Services Increased When Fees Were Removed: Lessons For Universal Health Coverage | No vaccination data |
| 41 | District-level health system strengthening for universal health coverage: evidence from a longitudinal cohort study in rural Madagascar, 2014-2018 | Is a review |
| 42 | Sex differences in health indicators among children in African DHS surveys | No vaccination data |
| 43 | Study Protocol: A Cross-Sectional Examination of Socio-Demographic and Ecological Determinants of Nutrition and Disease Across Madagascar | No vaccination data |
| 44 | [Circulation of the poliovirus in endemic zones with children vaccinated by the oral polio vaccine] | Unable to access full text |
| 45 | The impact of conflict on immunisation coverage in 16 countries | No sex-disaggregated vaccination data |
| 46 | Polio supplementary immunization activities and equity in access to vaccination: evidence from the demographic and health surveys | No sex-disaggregated vaccination data |
| 47 | Do equity funds protect the poor? Case studies from north-western Madagascar | No vaccination data |
| 48 | Data resource profile: WHO health equity monitor (HEM) | No sex-disaggregated vaccination data |
| 49 | Towards universal health coverage: the role of within-country wealth-related inequality in 28 countries in sub-Saharan Africa | Is a review |
| 50 | Huge poor-rich inequalities in maternity care: an international comparative study of maternity and child care in developing countries | Is a review |
| 51 | Contemporary epidemiological overview of malaria in Madagascar: operational utility of reported routine case data for malaria control planning | No sex-disaggregated vaccination data specific to Madagascar |
| 52 | Characterization of immunization secondary analyses using demographic and health surveys (DHS) and multiple indicator cluster surveys (MICS), 2006–2018 | Is a review |
| 53 | Gender and Immunization abridged report | No defined sample group |
| 54 | Determinants of health in developing countries: Cross-country evidence | No vaccination data specific to Madagascar |
| 55 | Global strategy for women’s and children’s health | No vaccination data |
| 56 | Contribution of integrated campaign distribution of long-lasting insecticidal nets to coverage of target groups and total populations in malaria-endemic areas in Madagascar | No vaccination data specific to Madagascar |
| 57 | Inclusion of gender and equity in maternal, newborn and child health services in West Africa: a literature review of programming | Only included in full text because could not identify abstract |
| 58 | Epidemiology and prevention of human papillomavirus and cervical cancer in sub‐Saharan Africa: a comprehensive review | Is a review |
| 59 | Basic health care provision and under-5 mortality: a cross-national study of developing countries | No vaccination data specific to Madagascar |
| 60 | Seasonal gaps in measles vaccination coverage in Madagascar | Is a review |
| 61 | Intra-household allocation of food and health care: current findings and understandings--introduction | No vaccination data |
| 62 | [Hepatitis B virus infection: a public health problem in Madagascar] | Unable to access full text |
| 63 | Baseline population health conditions ahead of a health system strengthening program in rural Madagascar | No sex-disaggregated vaccination data |
| 64 | Providing family planning services to remote communities in areas of high biodiversity through a Population-Health-Environment programme in Madagascar | No sex-disaggregated vaccination data |
| 65 | Action needed now to prevent further increases in measles and measles deaths in the coming years | No vaccination data |
| 66 | Inequities in on-time childhood vaccination: evidence from Sub-Saharan Africa | Duplicate data source |
| 67 | Measles outbreak in 2018-2019, Madagascar: epidemiology and public health implications | No sex-disaggregated vaccination data |
| 68 | Access to health care in developing countries: breaking down demand side barriers | No vaccination data |
| 69 | World health statistics 2009 | No sex-disaggregated vaccination data |
| 70 | Handbook on health inequality monitoring: with a special focus on low-and middle-income countries | No sex-disaggregated vaccination data specific to Madagascar |
| 71 | State of inequality: reproductive maternal newborn and child health: interactive visualization of health data | No sex-disaggregated vaccination data specific to Madagascar |
| 72 | Family and Reproductive Health Cluster biennial report: highlights of achievements in 2016-2017 | No sex-disaggregated vaccination data specific to Madagascar |
| 73 | Working together: an integration resource guide for immunization services throughout the life course | No sex-disaggregated vaccination data |
| 74 | Report of the Global conference on primary health care: from Alma-Ata towards universal health coverage and the Sustainable Development Goals | Is a review |
| 75 | The impact of vaccination on gender equity: conceptual framework and human papillomavirus (HPV) vaccine case study | No vaccination data specific to Madagascar |
| 76 | Reemergence of recombinant vaccine-derived poliovirus outbreak in Madagascar | Vaccination data is specific to a controlled trial |
| 77 | Study of the BCG Vaccine-Induced Cellular Immune Response in Schoolchildren in Antananarivo, Madagascar | Vaccination data is specific to a controlled trial |
| 78 | Etiologies, Risk Factors and Impact of Severe Diarrhea in the Under-Fives in Moramanga and Antananarivo, Madagascar | Vaccination data is specific to a controlled trial |
| 79 | Moramanga Health Survey, Madagascar | No sex-disaggregated vaccination data |
| 80 | Inequalities in cause-specific mortality in children and adolescents in the Moramanga health survey, Madagascar | No vaccination data |
| 81 | Cohort Profile: Moramanga health survey in urban and rural areas in Madagascar (MHURAM project) | No sex-disaggregated vaccination data specific to Madagascar |
| 82 | Assessment of poliovirus antibody seroprevalence in high risk areas for vaccine derived poliovirus transmission in Madagascar | Vaccination data is specific to a controlled trial |
| 83 | Investing in the health of girls and women: a best buy for sustainable development | No vaccination data |
| 84 | Health, Nutrition, and Population in Madagascar, 2000-09 | No sex-disaggregated vaccination data specific to Madagascar |
| 85 | Madagascar 2018-2019 measles outbreak response: main strategic areas | No sex-disaggregated vaccination data |
| 86 | Complete basic childhood vaccination and associated factors among children aged 12–23 months in East Africa: a multilevel analysis of recent demographic and health surveys | No sex-disaggregated vaccination data specific to Madagascar |
| 87 | Progress for children: achieving the MDGs with equity | Is an infographic |
| 88 | 40th anniversary of introduction of Expanded Immunization Program (EPI): a literature review of introduction of new vaccines for routine childhood immunization in Sub-Saharan Africa | Is a review |
| 89 | Commentary Child Immunization Cards: Essential Yet Underutilized in National Immunization Programmes | No vaccination data |
| 90 | Child health: reaching the poor | No vaccination data |
| 91 | Summary indices for monitoring universal coverage in maternal and child health care | No sex-disaggregated vaccination data specific to Madagascar |
| 92 | Individual and contextual factors associated with low childhood immunisation coverage in sub-Saharan Africa: a multilevel analysis | No sex-disaggregated vaccination data specific to Madagascar |
| 93 | Decomposing the rural-urban gap in the factors of under-five mortality in sub-Saharan Africa? Evidence from 35 countries | No vaccination data |
